# Reversibly Switching Silver Hierarchical Structures via Reaction Kinetics

**DOI:** 10.1038/srep14942

**Published:** 2015-10-07

**Authors:** Jianmei Liu, Tao Yang, Chengxiang Li, Jinhui Dai, Yongsheng Han

**Affiliations:** 1 Institute of Materials Science and Engineering, Ocean University of China, Qingdao, 266100, China; 2 State Key Laboratory of Multiphase Complex Systems, Institute of Process Engineering, Chinese Academy of Sciences, Beijing, 100190, China; 3 University of Chinese Academy of Sciences, Beijing, 100049, China

## Abstract

Here we report a study on controllable synthesis of hierarchical silver structures via regulating reaction kinetics. Silver particles with various morphologies are synthesized by a solution-based reduction approach at the addition of amino acids. The amino acid is used to coordinate with silver ions to slow down the reduction of silver ions. With the increase of glycine concentration, the morphologies of silver particles switch from dendrites, to flowers and to compacted spheres, which is attributed to the decrease of reaction rate as a result of the coordination. Three more amino acids are examined and confirms the role of reaction kinetic in shaping silver particles. Furthermore, by increasing the concentration of the reductant, the silver morphologies change from compact spheres to loose flowers as a result of the increase of reaction rate. Therefore the silver hierarchical structure can be reversibly switched by reaction kinetics. The silver particles synthesized are tested for surface enhanced Raman scattering (SERS) property and the dendritic particles present a remarkable SERS activity. This study shows that reaction kinetics is a powerful tool to tune hierarchical structures of silver particles, which is expected to be transferable to other material systems.

Silver is a promising material with the characters of low cost and high conductivity. It has been widely used in biological labeling and sensing, catalysis, conductors, localized surface plasmon resonance (LSPR), and surface-enhanced Raman scattering (SERS)^1^,^2^,^3^,^4^,^5^,^6^. Silver nanostructures with various shapes, such as wires, chains, belts, rice, plates, prisms, bars and cubes, have been synthesized by different approaches^7^,^8^,^9^,^10^,^11^,^12^,^13^,^14^,^15^,^16^,^17^. Sophisticated structures, such as flower-like, dendritic or star-shaped hierarchical silver particles are highly desired for improved SERS applications because these structures show significant electric field enhancement at their surface^18^,^19^. Owing to the complexity of hierarchical structures and the mystique of material formation process, controllable synthesis of hierarchical structures is still a challenge. Chemists have successfully controlled the structure of materials by employing capping agents and templates, but their success cannot be transferred to the cases without additives. Therefore, to controllable synthesis of hierarchical structures, we have to discover the mechanism dominating the structure development of materials.

Generally speaking, hierarchical structures are not thermodynamically favored shape but should be the products of kinetics. Among the kinetic factors, diffusion and reaction rates are two general parameters influencing the structure development of materials^20^,^21^. According to the Diffusion Limited Aggregation (DLA) model, a compact structure is normally formed under reaction limitation while a loose structure is prone to form at diffusion limited conditions^22^,^23^,^24^,^25^. By regulating diffusion and reaction rates, we have synthesized calcium carbonate particles with various morphologies^26^. To determine the role of diffusion and reaction separately, we have designed reactors to regulate the diffusion of chemicals, which leaded to the morphologies of platinum products switching from cubic to spherical^27^.

In this paper, we aim to discover the role of reaction kinetics in shaping materials. We synthesize silver particles by a solution-based reduction approach. Hydroxylamine is used as the reductant to reduce silver ions producing silver particles. The reduction is carried out in a solution containing amino acids which coordinate with silver ions to slow down the reduction. The reduction rate is regulated by changing the concentration of amino acids. By regulating the ratio of reductant and amino acids, the reaction rate can be reversibly switched, which leads to a reversible change of silver hierarchical structures from dendrites to spheres. The SERS activity of silver particles with diverse morphologies is investigated and the silver dendrites show excellent SERS enhancement.

## Results

**Figure 1:**
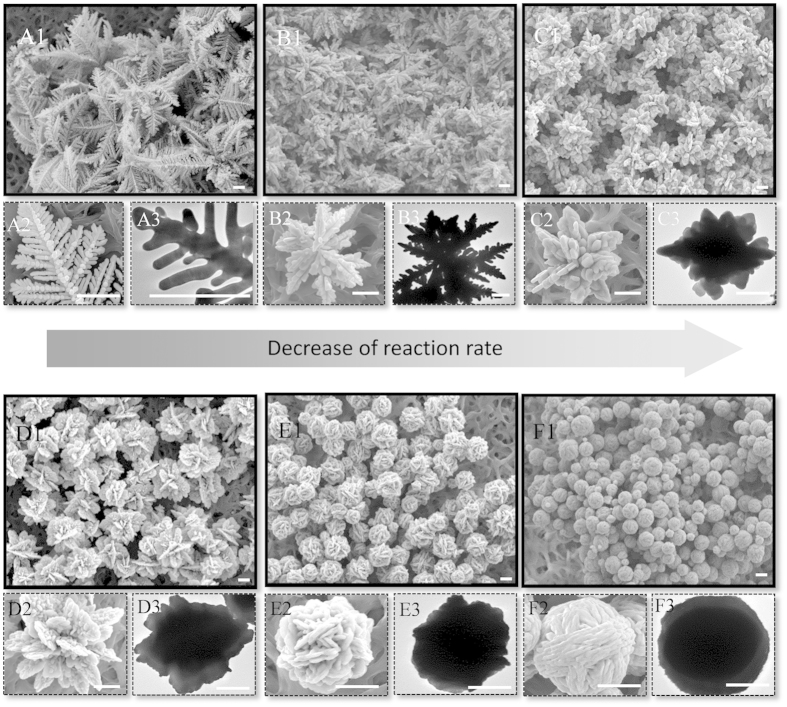
Electron microscopic images of silver products synthesized at different concentrations of glycine. (**A1**–**A3**) showing dendritic structure synthesized in a solution without glycine; (**B1**–**B3**) showing dendritic aggregates synthesized at 0.1 mM glycine; (**C1**–**C3**) showing radical aggregates synthesized at 1 mM; (**D1**–**D3**) showing flower like structures synthesized at 10 mM glycine; (**E1**–**E3**) showing flower bud-like structure synthesized at 20 mM; (**F1**–**F3**) showing sphere structure synthesized at 40 mM glycine. (**A3,B3,C3,D3,E3,F3**) are images taken by transmission electron. The scale bars represent 1 μm.

**Figure 2:**
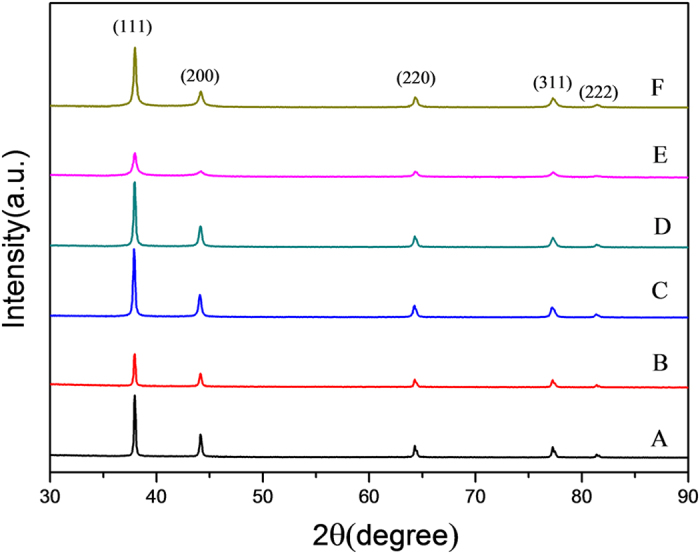
XRD patterns of silver particles synthesized at various concentrations of glycine. Samples (**A–F**) are synthesized at the addition of glycine in 0, 0.1, 1, 10, 20, 40 mM, respectively. Although the silver products synthesized at various concentration of glycine have different morphologies, their crystal structures and the main composition are similar.

The silver particles synthesized at various concentrations of glycine are characterized by electron microscopy, as shown in Fig. 1. Without the addition of glycine, leaf-like silver dendrites are largely formed, as shown in A1 of Fig. 1. A2 are magnified SEM image and TEM image of silver dendrites, respectively, which show that the silver dendrites are composed of main trunks and side branches. The structure description of silver dendrites and discussion on their formation have been reported previously^28^. Here we focus on the variation of dendrites after the addition of glycine. When 0.1 mM glycine is added into the solution, the dendrites become short and compact. Some dendrites jointly grow, forming dendritic aggregates, as shown in B1–B3 of Fig. 1. The increase of glycine concentration to 1 mM leads to the formation of radical aggregates, with the loss of dendritic feature, as shown in C1–C3 of Fig. 1. When the concentration of glycine is increased to 10 mM and 20 mM, flower-like particles composed of nanoplates are obtained, as shown in D1–D3 and E1–E3 of Fig. 1. The flowers in D1 largely exposed plate surfaces while the flowers in E1 become compact. When the concentration of glycine is increased to 40 mM, the silver products become compact spheres with diameter less than 2 micrometers. The crystal phase and composition of all silver products were characterized by X-ray diffraction (XRD), as shown in Fig. 2. All samples synthesized at various concentrations of glycine have the same diffraction spectra, which accords with the face centered cubic structure (JCPDS file No. 04–0783). There are no peaks of glycine due to the trace amounts of glycine involving in the silver particles.

The influence of glycine on the formation of hierarchical nanostructures has been investigated for a while but the mechanism is still unclear^29^,^30^,^31^. Some groups propose that glycine is a directing agent which guides the formation of hierarchical structures^19^. Others put forward that glycine selectively adsorbs on the facet, like other amino acids, leading to the formation of anisotropic structures^32^,^33^. In the glycine molecule there are many functional groups, such as −NH_2_ and −COOH, that have a strong tendency to coordinate with silver ions to form a stable complex with a stability constant of 7.76 × 10^6^ 34,35,36. Therefore, in the solution containing silver ions and glycine, there is a dynamic balance between silver ions and the complex. The addition of a reductant leads to a reduction of silver ions released from the complex forming silver products. Increasing the concentration of amino acid, more complexing agents surround silver ions and consequently silver ions are more likely to be coordinated than to be reduced by hydroxylamine, which results in a depression of the reduction. According to the RLA model, compact structures are prone to form under reaction-controlled condition^37^,^38^. Therefore, we assume that the morphology change of silver particles from dendrites to compact structures is mainly caused by the decrease of reaction rate.

**Figure 3:**
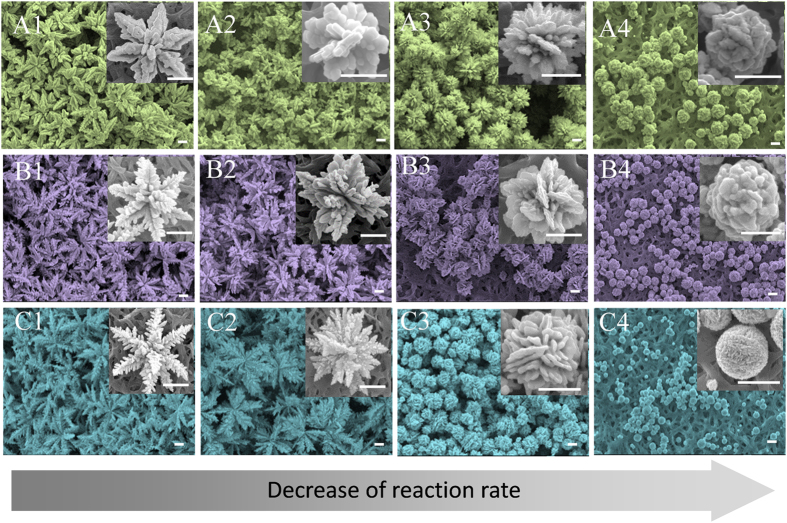
SEM images of silver products synthesized in arginine (A), leucine (B) and phenylalanine (C) solutions at various concentrations. (**A1**–**A4**) show the silver samples synthesized in arginine solution at 0.01, 1, 5, 10 mM, respectively; (**B1**–**B4**) show the silver samples synthesized in leucine solution at 0.01, 0.05, 1, 10 mM, respectively; (**C1**–**C4**) show the silver samples synthesized in phenylalanine solution at 0.01, 0.05, 0.1, 1 mM, respectively. The scale bars represent 1 μm.

To verify that the change of silver morphologies are caused by reaction rate instead of chemical template and selective adsorption, we synthesize silver products in different types of amino acid solution. Fig. 3 shows the morphologies of silver products synthesized at arginine (A), leucine (B) and phenylalanine (C) solutions. At each amino acid solution, with the increase of amino acid concentration, the silver products switch from dendritic aggregates to flower like particles and to spherical compact particles, which agrees well with the morphological change in the glycine system. Because the amino acids used in Fig. 3 have more functional groups and higher complex stability, the concentration of amino acid was varied from 0.01 mM and up to 10 mM. Further increase of the concentration does not change the spherical morphology very much, but makes the morphology more compact. Therefore, the morphological change of silver products is dependent on the concentration of amino acid but has little dependence on the types of amino acids, which excludes the role of chemical template in shaping particles. It also suggests that the reaction kinetics plays an important role in switching the morphologies of silver products.

**Figure 4:**
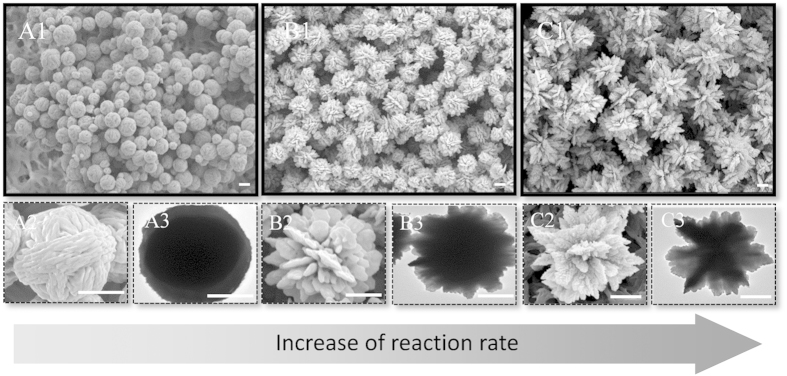
Electron microscopic images of silver particles synthesized at various concentration of hydroxylamine. (**A1**–**A3**) show silver particles synthesized at 10 mM hydroxylamine; (**B1**–**B3**) shows silver particles synthesized at 15 mM hydroxylamine; (**C1**–**C3**) show silver particles synthesized at 40 mM hydroxylamine. The increase of hydroxylamine concentration leads to an increase of reaction rate, resulting in the switch of particle morphologies from compact spheres to dendritic aggregates. The scale bars represent 1 μm.

To further verify the dominant role of reaction kinetics in switching the morphologies of silver particles, we conducted one more series of experiments, in which the glycine keeps the highest concentration of 40 mM while the concentration of the reductant increases. The increase of reductant concentration is expected to tune the balance of the competition between complexation and reduction to the reduction side, leading to an enhancement of the reduction rate. The silver products synthesized at different concentration of hydroxylamine are shown in Fig. 4. With the increase of hydroxylamine concentration from 10 mM to 15 mM to 40 mM, the morphology of the products changes from compact spheres to flower like particles and to dendritic aggregates, respectively. These morphologies are similar with the structures shown in Fig. 1 and Fig. 3. But the morphologies change from compacted sphere to loose flowers, which is opposite to the change caused by the increase of glycine concentration. This is because the increase of glycine concentration leads to a decrease of reduction rate while the increase of hydroxylamine concentration results in an increase of reduction rate, which again confirms that the change of silver morphology is dominated by reaction kinetics. By changing the ratio of reductant and complex agent, the silver morphologies are reversibly switched.

**Figure 5:**
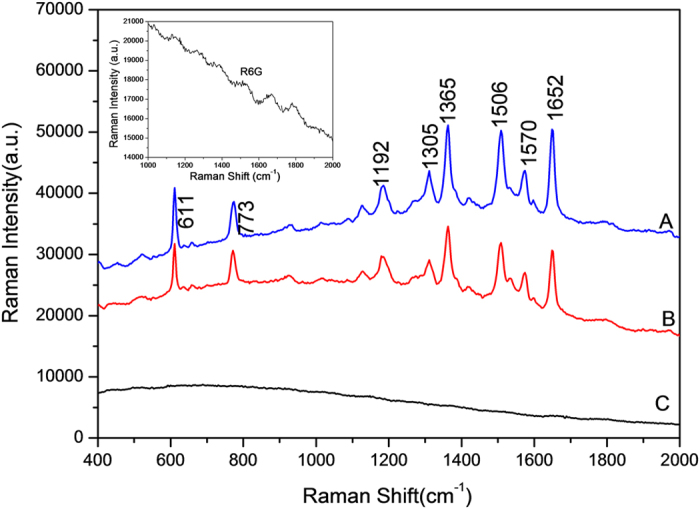
Surface-enhanced Raman scattering spectra of silver samples with diverse morphologies, (A) spectra from silver dendrites, (B) spectra from silver dendritic aggregates, (C) spectra from silver spheres. The insert is the Raman spectrum of free R6G.

The SERS sensitivity of the prepared silver particles was investigated by using Rhodamine 6G (R6G) as a probe molecule. The resulting SERS spectra of 5 μM R6G from silver dendrite (as shown in Fig. 1A), dendritic aggregates (as shown in Fig. 1B), spherical silver particles (as shown in Fig. 1F) are collected and shown in Fig. 5A-5C, respectively. Remarkable peaks are observed in the solution containing dendritic and aggregated silver particles, as shown in Fig. 5A-5B, while there are no peaks detected in the solution containing spherical silver particles, as shown in Fig. 5C. The Raman signals observed at 611 cm^−1^ is due to C-C-C in-of-plane transforming mode for R6G while 773 cm^−1^ can be assigned to C-H out-of-plane transforming. The band present at 1192 cm^−1^ is assigned to vibration of xanthene ring bending. The bands at 1305, 1570 cm^−1^are due to the stretching vibration of C = C and C = O, respectively. The bands present at 1365, 1506 and 1652 cm^−1^ are due to the stretching vibration of C-C of xanthene ring. All these detected peaks belong to the R6G^39^, as shown in the inset of Fig. 5.

Compared with the spectrum of free R6G, the spectra taken from dendritic and aggregated silver particles demonstrate a remarkable enhancement. The absolute value of the enhancement factor (EF) was calculated by using a formula, EF = *I*_SERS_/*I*_O_ × *N*_O_/*N*_SERS_^40^,^41^. *I*_SERS_ and *I*_O_ are the Raman intensity of the same Raman band under SERS and normal Raman conditions, respectively. *N*_O_ and *N*_SERS_ are the number of free molecules and adsorbed molecules for SERS, respectively. Here, the strongest Raman bands at 1652 cm^−1^ are selected to calculate the EF where the signal of substrate can be ignored. *N*_O_ is calculated on the basis of the estimated concentration of surface R6G. We used single R6G molecule coverage (2.22 nm^2^) reported in the literature to calculate the number of adsorbed R6G on the silver particles surface^42^. The intensities of the Raman peaks are enhanced by an EF of approximately 7.96 × 10^4^, 5.70 × 10^4^ for silver dendrites and dendritic aggregates particles, respectively. Therefore, the dendritic and aggregated particles demonstrate a significant SERS effect^43^,^44^. This is because these two loose structures not only generate abundant “hot spots” from the gaps formed by junctions but also provides a large surface area for the spatial loading molecules. Thus, the dendritic silver particles are good candidates to be active SERS substrates. More detailed work in this direction needs to be carried out in next steps.

## Discussion

To understand why the reaction kinetics dominates the morphology of silver products, we have to consider the formation mechanism of silver dendrites. The growth of dendrites is mainly determined by the chemical distribution around the growth front^45^. When a concentration gradient is present at the surface of growth front, protrusions are easily formed as a result of the instability of crystal surface. The initial protrusions later grow up forming dendritic structures. Therefore, the growth of dendritic structures is dependent on the chemical concentration around the growth front, which is determined by the diffusion and reaction rates of chemicals. In this paper, the increase of amino acid concentration leads to a decrease of reaction rate, which slows down the consumption of chemicals, leading to a tendency of homogeneous chemical distribution around the growth front. The homogeneous distribution disfavors the growth of dendritic structures. On the other hand, the increase of hydroxylamine concentration speeds up the reaction, which makes the consumption of chemicals faster than their supplement, creating chemical gradient around the growth front, thus promoting the formation of loosely dendritic silver structures. The formation of other hierarchical structures is a result of a competition of the dendritic growth and minimization of surface free energy. When the conditions do not favor the growth of dendritic structure, the initially-formed small dendrites cannot grow up quickly. They prefer to aggregate to minimize their surface. When the aggregation dominates the growth of silver particles, spherical compacted particles are formed as a result of the minimization of surface free energy. In the conditions between aggregation dominant and growth dominant, the compromise of these two competing growth processes leads to the formation of diverse hierarchical structures. Therefore, by regulating reaction kinetics, the growth process of silver particles can be changed, which results in a reversible switch of silver morphologies with high repeatability and high yield.

In summary, various hierarchical silver structures, including dendrites, flowers and spherical aggregates, were controllably synthesized by a solution based reduction approach at the addition of different amount of amino acids. The amino acids coordinate with silver ions to form a stable complex, which slowed down the reduction. With the increase of amino acid concentration, the reduction rate was reduced, leading to the morphologies of silver products switching from loose dendritic structures to compact spherical structures. Four types of amino acids were employed to regulate silver structures, which confirmed the role of amino acids. On the other hand, an increase of hydroxylamine concentration resulted in an enhancement of the reduction rate, which leaded to an opposite change of silver morphologies. Therefore, by regulating reaction kinetics, the hierarchical structures of silver products were reversibly switched. The SERS activity of the silver samples were examined and the dendritic structure presented a better result. This study showed that reaction kinetic is a powerful tool in shaping materials and a promising approach for controllable synthesis of hierarchical structures.

## Methods

All chemicals were of analytical grade and used without further purification. They were purchased from Sigma-Aldrich. Deionized water with a resistivity higher than 18.2 MΩ used throughout the experiments was generated by a Milli-Q system (Millipore, USA).

In a typical experiment, 2 mL silver nitrate (100 mM) and a defined amount of amino acid were dissolved in 40 ml deionized water, respectively, stirring for 10 minutes to form a stable complex. Then a designed amount of hydroxylamine solution (200 mM) was added into the above mixture to initiate the reduction. The reaction lasted for 30 minutes in a thermostatic water bath at 25 °C.

The morphologies of synthesized products were characterized by a JSM-7001F Thermal Field Scanning Electron Microscope (JEOL, Japan) and a JEM-2100 (UHR) Transmission Electron Microscope (JEOL, Japan) at an accelerating voltage of 200 kV. The phase and composition of the products were determined by X-ray Diffractometry (PANalytical B.V., Netherlands), using CuKα radiation, and the data were collected over the range of 2θ from 30° to 90°. Samples for SEM imaging were prepared by pipetting the product suspension onto an organic membrane. Samples for TEM analysis were prepared by dropping 10 μL product suspension onto carbon-coated copper TEM grids. Samples for SERS measurements were prepared by dropping a total of 100 μL of 0.5 mM silver suspension on the glass substrate. The silver suspension contains 5 μM Rhodamine 6G and 50 mM KCl. The substrate was dried and rinsed with deionized water for three times to remove free R6G molecules. For comparison purposes, a glass substrate covered with R6G molecules was prepared by directly dropping R6G solution on the glass substrate. All the Raman spectra were acquired for 10 s at a smart Raman spectrometer (LabRAM ARAMIS) under a 532 nm laser and a spot size of 1 μm for excitation.

## Additional Information

**How to cite this article**: Liu, J. *et al.* Reversibly Switching Silver Hierarchical Structures via Reaction Kinetics. *Sci. Rep.* **5**, 14942; doi: 10.1038/srep14942 (2015).
